# Nrf2 Is an Attractive Therapeutic Target for Retinal Diseases

**DOI:** 10.1155/2016/7469326

**Published:** 2016-10-12

**Authors:** Yasuhiro Nakagami

**Affiliations:** ^1^Daiichi Sankyo Co., Ltd., 1-2-58 Hiromachi, Shinagawa-ku, Tokyo 140-8710, Japan; ^2^Asubio Pharma Co., Ltd., 6-4-3 Minatojima-Minamimachi, Chuo-ku, Hyogo 650-0047, Japan

## Abstract

Nuclear factor erythroid 2-related factor 2 (Nrf2) is a redox-sensitive transcription factor that binds to antioxidant response elements located in the promoter region of genes encoding many antioxidant enzymes and phase II detoxifying enzymes. Activation of Nrf2 functions is one of the critical defensive mechanisms against oxidative stress in many species. The retina is constantly exposed to reactive oxygen species, and oxidative stress is a major contributor to age-related macular diseases. Moreover, the resulting inflammation and neuronal degeneration are also related to other retinal diseases. The well-known Nrf2 activators, bardoxolone methyl and its derivatives, have been the subject of a number of clinical trials, including those aimed at treating chronic kidney disease, pulmonary arterial hypertension, and mitochondrial myopathies. Recent studies suggest that Nrf2 activation protects the retina from retinal diseases. In particular, this is supported by the finding that Nrf2 knockout mice display age-related retinal degeneration. Moreover, the concept has been validated by the efficacy of Nrf2 activators in a number of retinal pathological models. We have also recently succeeded in generating a novel Nrf2 activator, RS9, using a biotransformation technique. This review discusses current links between retinal diseases and Nrf2 and the possibility of treating retinal diseases by activating the Nrf2 signaling pathway.

## 1. Retinal Diseases and Oxidative Stress

The free radical theory of aging was advocated in 1954 [1] and had a dramatic effect on contemporary medicine, including molecular biology. It was hypothesized that oxidative stress, especially within mitochondria, led to a vicious cycle in which cells are directly damaged. Although many researchers were initially reluctant to accept this concept, accumulating evidence has now consolidated a role for oxidative stress, which is exerted by the intracellular accumulation of reactive oxygen species (ROS) [2], in aging and many diseases. Retinal diseases are no exception to this concept. More than 99% of ultraviolet radiation is absorbed by the anterior segment of the eye and the crystalline lens, but the remaining 1% reaches the light-sensitive retina [3, 4]. Studies have linked the early development of age-related macular degeneration (AMD) with exposure to intense ultraviolet radiation or bright sunlight. Drusen deposits, which are initially formed by oxidized lipids, have been shown to be associated with AMD, along with laminar deposits in Bruch's membrane [5, 6]. It is also well known that the major lipofuscin fluorophore A2E mediates age-related pathophysiological processes in the retinal pigment epithelium (RPE) [7]. Light-induced A2E derivatives such as oxiranes and epoxides were shown to cause DNA damage and induce cellular death [8, 9]. Light emitting diode light was shown to result in cellular damage, which is wavelength-dependent in that short-wavelength light is not easily absorbed into the cornea and lens [10]. Hyperglycemia is also a critical factor in retinal degeneration, especially in diabetic macular edema (DME) [11, 12]. High glucose levels activate the polyol pathway and cause accumulation of advanced glycation end products (AGEs) and overactivation of hexosamine and protein kinase C (PKC) pathways that exaggerate inflammation. In many cases of DME, abnormal retinal neovascularization is observed, as is also seen in wet AMD, and the generation of ROS is indirectly involved in the pathogenesis. The final common pathway in a variety of retinal diseases, including glaucoma and retinitis pigmentosa, is linked to retinal cellular death, and it is likely that ROS triggers cellular damage in these cases. Factors such as an abundance of polyunsaturated lipids, high oxygen consumption ratio, hypoxia, psychological stress, radiation, air pollution including ozone, smoking, and oxidized foods are other typical risk factors that can induce the generation of ROS in the retina [13–16].

Based on the relationship between retinal diseases and oxidative stress, vitamins and minerals have been regarded as a promising preventive remedy, especially for dry AMD. The Age-Related Eye Disease Study (AREDS) is a major clinical trial sponsored by the National Eye Institute to investigate the effects of vitamin C, vitamin E, beta-carotene, and zinc. AREDS2 is a multicenter, randomized trial designed to investigate the effects of adding macular xanthophylls (lutein and zeaxanthin) and/or long-chain omega-3 fatty acids (docosahexaenoic acid [DHA] and eicosapentaenoic acid [EPA]) to the original AREDS formula on the progression to advanced AMD [17]. These supplements did appear to reduce the risk of progression towards advanced AMD, but the effects were not significant. Results using other antioxidants such as alpha-tocopherol, nicanartine [11], N-acetyl-L-cysteine [18], vitamin A [19], and OT-551 [20] were disappointing or remain controversial, questioning the validity of antioxidants as a therapeutic target for retinal diseases. However, the extremely short half-life and weak activity of standard radical acceptors remain critical issues that should be considered in future clinical trials.

## 2. Oxidative Stress and Nrf2

**Figure 1:**
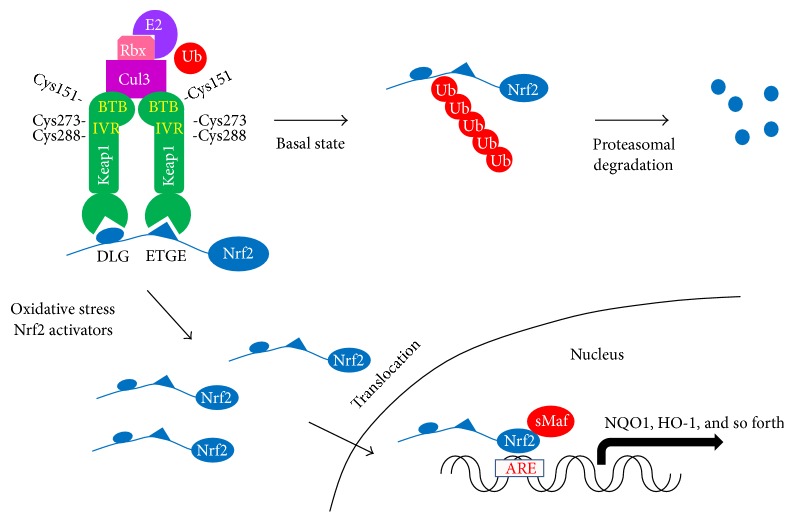
Keap1-Nrf2 signaling. The Keap1 homodimer binds to a single Nrf2 molecule via DLG and ETGE motifs; the latter has a high affinity for Nrf2. Under basal conditions, the Keap1-Nrf2 complex is degraded by Cul3-dependent E3 ubiquitin ligase. However, Keap1 also works as a stress sensor, and oxidative stress induces conformational changes of Keap1-Nrf2 complex. As a result, ubiquitination of Nrf2 is inhibited, and then stabilized Nrf2 is translocated into the nucleus. Binding of Nrf2 with small Maf proteins to the antioxidant response element (ARE) induces many antioxidant and phase II detoxifying enzymes.

Nuclear factor erythroid 2-related factor 2 (Nrf2), which belongs to the basic leucine zipper (bZIP) transcription factor and heterodimerizes with small Maf proteins, functions as a key player in the redox homeostatic gene regulatory network. The expression of Nrf2 is observed in many tissues, particularly those exposed to the external environment (skin, lungs, and gastrointestinal tract) and those associated with detoxification (liver and kidneys) [21]. Under resting conditions, Nrf2 is trapped within the cytosol by an adaptor protein, Kelch-like erythroid cell-derived protein with CNC homology- (ECH-) associated protein 1 (Keap1) (Figure 1). This protein is an adaptor component of Cullin 3- (Cul3-) based ubiquitin E3 ligase; therefore Nrf2 is rapidly degraded via the proteasomal pathway. Several cysteine residues within Keap1 serve as primary sensors of stress signals, and their modification leads to conformational changes in Keap1, thereby inhibiting ubiquitination of Nrf2 [22]. As a result, stabilized Nrf2 is translocated into the nucleus. Nrf2 binds to a specific consensus cis-element, named the antioxidant-response element (ARE; also called the electrophile-responsive element [EpRE]), present in the promoter region of genes encoding antioxidant and phase II detoxifying enzymes. There are over 250 Nrf2-targeted genes, including NAD(P)H:quinone oxidoreductase-1 (NQO1), heme oxygenase-1 (HO-1), glutamate cysteine ligase, glutathione S-transferase, glutathione peroxidase, catalase, superoxide dismutase, and thioredoxin UDP-glucuronosyltransferase [23]. Therefore, it is predicted that driving robust expression of these genes would be activated by Keap1-Nrf2 signaling, which would rescue cells from a variety of stimuli such as reactive toxicants, proinflammatory factors, apoptosis, and carcinogenesis [22]. Among them, the potential crosstalk between the nuclear factor-*κ*B (NF-*κ*B) and Nrf2 pathways remains still unclear [24].

## 3. Nrf2 and Retinal Diseases

Oxidative stress is one of the main causes of the pathogenesis of chronic obstructive pulmonary disease (COPD) [25]. Cigarette smoke directly damages the alveolar epithelium and lung structures, leading to deterioration of lung function. As a result, targeting oxidative stress has been intensively studied in the pulmonary field, and accumulating evidence from patients with COPD suggests a role for Nrf2 in this process. The level of Nrf2 mRNA in lavaged macrophages of young patients is not dependent on smoking status; however, levels were reduced in samples from old current smokers compared to old nonsmokers [26]. These observations strengthen the idea that the aging process weakens Nrf2 activity. In the ocular field, a decrease in Nrf2 DNA-binding activity was reported in the retina of patients with diabetic retinopathy [27], and a role for Nrf2 in the onset of AMD has been well documented [15, 28]. These data identify either inhibiting the decrease or activating Nrf2 as potential strategies for treating retinal diseases. This approach could be suitable for scavenging ROS and suppressing retinal degeneration. In addition, it seems Nrf2 is highly expressed in the ganglion cell layer and the inner nuclear layer with the strong expression of Muller cells [29]. Further investigations to scrutinize the expressional change of Nrf2 in pathological conditions are important to establish appropriate remedies.

Data from Nrf2 knockout mice represent the rationale for the concept of Nrf2-mediated cellular protection. Although Nrf2 is considered one of the master genes, Nrf2 knockout mice develop without critical deficits and reach adulthood [30, 31]. This indicates that Nrf2 is dispensable for development and/or that its absence may be compensated for by Nrf2-like genes. However, the ocular phenotypes seen in these mice are exacerbated under oxidative circumstances. Age-dependent deterioration in the RPE, including accumulation of lipofuscin and drusen-like deposits and spontaneous choroidal neovascularization (CNV), was observed in Nrf2 knockout mice compared to wild-type controls [32]. Cigarette smoke induced more profound damage to the RPE-Bruch membrane in Nrf2 knockout mice, where large cytoplasmic vacuoles were observed [33]. Retinal degeneration in these mice is exacerbated under many pathological conditions including retinopathy of prematurity (oxygen-induced retinopathy) [34], glaucoma (axonal injury by nerve crush) [35], diabetic retinopathy [29], posterior uveitis [36], and central retinal artery occlusion (retinal ischemia-reperfusion) [37]. In addition to oxidative stress and inflammation, visual dysfunction, retinal thickness, leukocyte adherence, and cellular survival are employed as surrogate markers to evaluate the retinal deterioration. Such preclinical data sets are motivating researchers to work towards identifying novel Nrf2 activators.

Keap1 knockout mice were generated with the expectation that Nrf2-targeted genes would be increased and the mice would become resistant to oxidative stress [38]. However, these mice died before weaning due to hyperproliferation of keratinocytes in the esophagus and forestomach, resulting in an abnormal upper digestive tract. In a subsequent study, Keap1 was disrupted in a hepatocyte-specific manner, and these homozygous mice showed no deficits in the development or physiological integrity of the liver. Moreover, the mice showed chronic activation of Nrf2 and resistance to acetaminophen-induced hepatotoxicity [39]. These results strongly support the concept that tissue-specific activation of Nrf2 would be an ideal therapy for treating retinal diseases, without causing severe adverse effects.

## 4. Nrf2 Activators Ameliorate Oxidative Stress-Related Retinal Diseases

**Figure 2:**
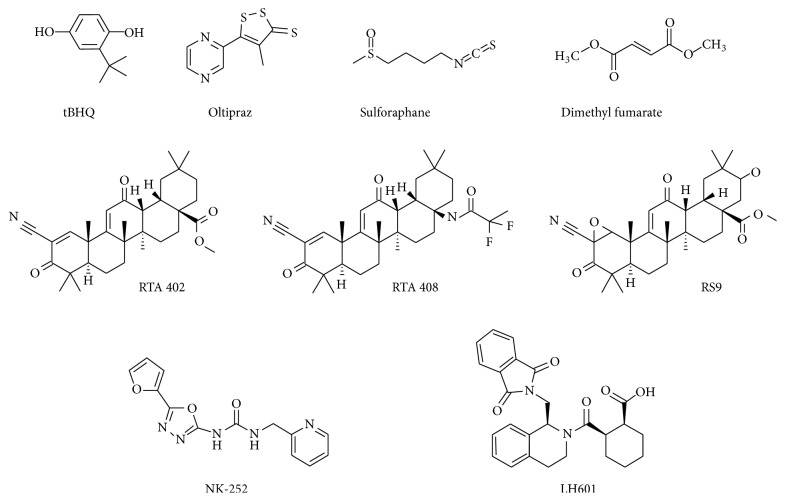
Chemical structure of typical Nrf2 activators.

Sulforaphane, which is found in broccoli sprouts and other cruciferous vegetables, has long been employed as a benchmark Nrf2 activator [40] (Figure 2). This compound is a weak electrophile that has the ability to react with cysteine thiols of Keap1 [41]. The* in vitro* activity of sulforaphane is generally determined by the concentration required to double NQO1 activity (CD) in murine Hepa1c1c7 hepatoma cells. From this, the CD value of sulforaphane is known to be approximately 200 nM. Similar well-known electrophiles include curcurmin, resveratrol (a polyphenolic compound found in mulberries, grapes, and red wine), oltipraz, ebselen, tertiary-butylhydroquinone (tBHQ), and dimethyl fumarate (also called BG-12 or Tecfidera), some of which have undergone clinical trials [42]. Despite the fact that not all of them show low CD values [43], it is worth noting that dimethyl fumarate has been approved by the FDA as a new oral drug treatment for patients with relapsing-remitting multiple sclerosis [44]. Because the CD value of dimethyl fumarate is over 10 *μ*M, Nrf2-independent modes of action also seem to be involved in its efficacy, including the activation of hydroxycarboxylic acid receptor 2 (HCAR2) and immunomodulatory properties.

A breakthrough in the study of Nrf2 activators was the finding that some synthetic oleanane triterpenoids have CD values in the subnanomolar range [45, 46]. Within this context, a variety of triterpenoids have been chemically synthesized and intensively tested over a long period. The best-known triterpenoid is bardoxolone methyl [2-cyano-3,12-dioxooleana-1,9(11)-dien-28-oic acid (CDDO) methyl ester/CDDO-Me/RTA 402/bard], which has a CD value of approximately 1 nM, and is classified as an oral “antioxidant inflammation modulator.” Clinical data from a phase 2b trial were partially reported in 2011 [47]. In the BEAM study (“Bardoxolone Methyl Treatment: Renal Function in Chronic Kidney Disease/Type 2 Diabetes”), the safety and efficacy of RTA 402 in patients with moderate to severe chronic kidney disease associated with type 2 diabetes (227 patients, 52 weeks) were investigated. RTA 402 improved the estimated glomerular filtration rate at 24 weeks and this efficacy continued to 52 weeks. But the phase 3 trial, called the BEACON study (“Bardoxolone Methyl Evaluation in Patients with Chronic Kidney Disease and Type 2 Diabetes: The Occurrence of Renal Events”) (1600 patients with Stage 4 chronic kidney disease), was to be terminated in 2012 because of a higher rate of cardiovascular mortality [48]. It seems that volume retention and acute sodium were the cause of these cardiovascular events [49]; however, the controversy has not been fully cleared as to whether both efficacy and adverse events were the result of Nrf2 activation [50]. RTA 402 is thus undergoing further evaluation regarding its use in the treatment of chronic kidney disease and pulmonary hypertension [42].

Oleanolic acid is commonly used as the starting material for synthetic oleanane triterpenoids, and modification at position C17 is often used to synthesize CDDO scaffolds such as imidazole (CDDO-Im), methyl amide (CDDO-MA), ethyl amide (CDDO-EA), trifluoroethyl amide (CDDO-TFEA), nitrile (di-CDDO), and difluoro-propanamide (RTA 408/omaveloxolone). In addition, RTA 408 is currently being tested in clinical trials into postsurgical corneal endothelial cell loss (https://clinicaltrials.gov/; Identifier: NCT02128113) [42].

The triterpenoids described above have been also employed in preclinical experiments and have been shown to be active in several ocular models. Light damage causes an increase in retinal oxidative stress immediately following exposure, and this has been widely used to study the mechanisms of age-related retinal cellular death. When mice were fed CDDO-TFEA, the thinning of the outer nucleus layer was suppressed [51]. The ratio of CDDO-TFEA in their diet was 100–200 mg/kg, which is approximately equal to oral administration of 0.1–0.2 mg/kg. Optical nerve crush injury is commonly used as an experimental disease model for glaucoma and traumatic optic neuropathy. Oral administration of 16 mg/kg CDDO-Im attenuated the death of retinal ganglion cells in mice [35]. Endotoxin-induced uveitis is one of several animal models of ocular inflammation that is triggered by lipopolysaccharide. Ocular inflammatory responses, including leukocyte adherence to retinal vasculature, were decreased by intraperitoneal pretreatment with CDDO-Im at a dose of 1.6 mg/kg [36]. Retinal capillary degeneration following ischemia-reperfusion was also markedly attenuated when mice were intraperitoneally pretreated with 0.5 mg/kg RTA 402 [37]. In the latter three experiments, neither CDDO-Im nor RTA 402 was effective in Nrf2 knockout mice, indicating that the mechanism of triterpenoid function is dependent on Nrf2 activation. It is likely that the effective oral dose range is approximately 1–10 mg/kg, which seems rational considering the dose of 20 mg/kg used in the BEACON study [48]. However, because a preventive protocol and acute injury were employed in these experiments, their effects should be also investigated using therapeutic protocols and/or in chronic disease models. Exposure to cigarette smoke for 6 months induced alveolar apoptosis, alveolar destruction, and pulmonary hypertension, and this damage, including lung oxidative stress, was inhibited when mice were fed a diet containing CDDO-Im (60 or 90 mg/kg) [52]. These results imply that triterpenoids could also be effective in chronic retinal disease models.

We recently identified a novel potent Nrf2 activator, RS9 (CD value: 0.2 nM), using a biotransformation technique. Using* in vivo* studies we demonstrated that oral administration of RS9 inhibits neovascularization in oxygen-induced retinopathy in rats, and an intravitreal injection of RS9 suppressed blood-retinal barrier permeability in glycated albumin-injected rabbits [53]. The chemical structure of RS9 is unique because it contains epoxidation in the A-ring and hydroxylation in the E-ring. From our structure-activity relationship study, it is likely that the epoxidation contributes to a reduction in cytotoxicity and the hydroxylation is involved in the improvement of activity. The efficacy of RS9 was also confirmed in rhodopsin Pro347Leu transgenic rabbits, which were previously generated as a model of retinitis pigmentosa [54]. Intravitreal injections of RS9 microspheres were employed in this study, but small implants that release more compounds could be a more realistic method for future clinical trials.

Although both preclinical and clinical data have suggested the therapeutic potential of Nrf2 activators for treating retinal diseases, selection of the best diseases remains to be discussed. Because continuing oxidative stress damages the retina over time and activation of Nrf2 is not expected to reverse cellular death, preventing the progress of dry AMD might be a more promising target, as in the case of the AREDS study. Systemic adverse events are still a big concern of triterpenoids; therefore, administering them via eye drops or intraocular implants would be a promising approach for lowering these risks [55, 56].

## 5. Novel Strategies for Activating Nrf2

Human Keap1 is a 70 kDa cysteine-rich protein (624 amino acids and 27 cysteines), of which residues Cys 151, 273, and 288 have been shown to be essential for regulating Nrf2 in biological studies. Most of the currently used Nrf2 activators are electrophiles and are thought to form covalent adducts with the sulfhydryl groups by Michael addition reactions on the specific cysteine residues of Keap1. Higher concentrations of these compounds interact with other cysteine-rich proteins with lower binding affinities, leading to activation of caspases and induction of apoptosis [46]. To overcome this issue, peptide and small molecule inhibitors of the Keap1-Nrf2 protein-protein interaction (PPI) have been investigated [57, 58]. The Keap1 homodimer binds to Nrf2 through a “hinge and latch” mechanism, in which the ETGE and DLG motifs of Nrf2 interact with Keap1-DC domains (the double glycine repeat domain [DGR] plus the C-terminal region of Keap1) [59]. X-ray crystallography data suggest that the druggability of the interface is not low. The peptide mimetics of the ETGE and DLG motifs are therefore expected to be effective, although it is still important to solve peptide-related issues, such as cellular permeability and plasma stability. Several chemical compounds have been identified from high-throughput screening, and some of them, such as LH601A, have been shown to accelerate the translocation of Nrf2 to the nucleus [60]. Few studies into PPI inhibitors of the Keap-Nrf2 interaction have demonstrated* in vivo* efficacy, but NK-252 (1-(5-(furan-2-yl)-1,3,4-oxadiazol-2-yl)-3-(pyridin-2-ylmethyl)urea) has been shown to interact directly with Nrf2-containing Keap1-DC domains. Furthermore, oral administration of NK-252 inhibits the progression of nonalcoholic steatohepatitis-related fibrosis in rats at a dose of 20 or 60 mg/kg [61]. This study used a weak electrophile, oltipraz, for comparison, which has since been tested for liver fat reduction in patients with nonalcoholic fatty liver disease, excluding liver cirrhosis (https://clinicaltrials.gov/; Identifier: NCT02068339). It will be intriguing to determine whether the effect of NK-252 is diminished in Nrf2 knockout mice, whether efficacy is observed in retinal disease models, and whether NK-252 shows acceptable tolerability.

Keap1 functions as an adaptor protein for the regulation of proteasomal degradation of Nrf2 by Cul3-dependent E3 ligase. Therefore inhibiting the interface between Keap1 and Cul3 represents another approach for activating Nrf2 [62]. In this context, the intervening-region (IVR) domain [63] and the Broad complex, Tramtrack and Bric-à-Brac (BTB) domains [64] of Keap1 have been studied using experimental deletion. Because many protein-protein interfaces are flat and large, confirming the existence of suitable drug target sites in the ubiquitin-proteasome system will be essential before drug candidates can be identified. However, the X-ray structure of the Keap1-Cul3 interaction is still unknown. Such an analysis could identify a number of promising compounds that act to inhibit the Keap1-Cul3 interface, such as the proteasome inhibitor bortezomib (marketed as Velcade for the treatment of multiple myeloma).

Small interfering RNA (siRNA) is a breakthrough technology used to silence genes by causing destruction of specific mRNA molecules. Considering the mechanisms of Keap1-Nrf2 signaling, Keap1 siRNA is predicted to be a highly successful strategy to specifically potentiate Nrf2 signaling. The half-life of Nrf2 in peritoneal macrophages is estimated to be 18.5 minutes [65], indicating that Nrf2 is constantly produced and rapidly degraded. This profile is suitable to the application of an siRNA-based method. Although there have been no reports to date that Keap1 siRNA is effective in* in vivo* retinal disease models, other siRNA-based drugs are being tested in clinical ophthalmological trials. QPI-1007 is new synthetic siRNA designed to inhibit caspase 2 expression and has been tested in patients with nonarteritic anterior ischemic optic neuropathy and other optic neuropathies [66]. PF-655 (REDD14NP/RTP801i) is an siRNA that targets the RTP801 (DDIT4/Redd1) gene, which is induced under hypoxia and cellular stress and inhibits the mTOR pathway. This siRNA has been evaluated for the treatment of wet AMD and diabetic macular edema [67]. It is possible that the results from these trials will encourage researchers to investigate the use of Keap1 siRNA to activate Nrf2 signaling in retinal disease models.

## 6. Concluding Remarks

Many questions remain unanswered regarding the mechanisms by which oxidative stress Nrf2 is involved in the onset of retinal diseases. However, activation of Nrf2 would be worth testing further using pathological models. With respect to clinical applications, the reversibility of Nrf2 activation should be considered. In this regard, RTA 402 and RTA 408 have been tested in clinical trials; however, the mode of action of Keap1 is Michael addition, which is known to be an irreversible process. Reducing these irreversible properties, using reversible compounds, or further improving selectivity might also be useful in order to avoid such adverse events as those observed in the BEACON study [48]. In addition, because cancer cells harness the aberrant activation of Nrf2 for proliferation and survival under stress conditions, carcinogenesis should be also studied in some detail [68]. To date, the efficacy of Nrf2 activators has been shown mostly by oral administration. Therefore, focal administration such as via eye drops or intravitreal injection could be one strategy that would minimize adverse systemic events during the treatment of ocular diseases. In terms of drug delivery to the retia, nanoparticle techniques may be appropriate for long-term treatment. Although* in vivo* pharmacological and toxicological studies will certainly be needed in the future, data are continuing to accumulate that are shedding light on the use of Nrf2 activation to treat oxidative stress-related retinal/retinovascular diseases. The success of these methods will depend on careful optimization of the compounds, appropriate selection of diseases, and use of a proper drug-delivery system to the retina.

## References

[1] Harman D. (1956). Aging: a theory based on free radical and radiation chemistry. *Journal of Gerontology*.

[2] DeBalsi K. L., Hoff K. E., Copeland W. C. (2016). Role of the mitochondrial DNA replication machinery in mitochondrial DNA mutagenesis, aging and age-related diseases. *Ageing Research Reviews*.

[3] Glickman R. D. (2011). Ultraviolet phototoxicity to the retina. *Eye and Contact Lens*.

[4] Majdi M., Milani B. Y., Movahedan A., Wasielewski L., Djalilian A. R. (2014). The role of ultraviolet radiation in the ocular system of mammals. *Photonics*.

[5] Crabb J. W., Miyagi M., Gu X. (2002). Drusen proteome analysis: an approach to the etiology of age-related macular degeneration. *Proceedings of the National Academy of Sciences of the United States of America*.

[6] Hammes H.-P., Hoerauf H., Alt A. (1999). N(*ε*)(carboxymethyl)lysin and the AGE receptor RAGE colocalize in age-related macular degeneration. *Investigative Ophthalmology & Visual Science*.

[7] Kim S. R., Jockusch S., Itagaki Y., Turro N. J., Sparrow J. R. (2008). Mechanisms involved in A2E oxidation. *Experimental Eye Research*.

[8] Sparrow J. R., Vollmer-Snarr H. R., Zhou J. (2003). A2E-epoxides damage DNA in retinal pigment epithelial cells. Vitamin E and other antioxidants inhibit A2E-epoxide formation. *The Journal of Biological Chemistry*.

[9] Radu R. A., Mata N. L., Bagla A., Travis G. H. (2004). Light exposure stimulates formation of A2E oxiranes in a mouse model of Stargardt's macular degeneration. *Proceedings of the National Academy of Sciences of the United States of America*.

[10] Kuse Y., Ogawa K., Tsuruma K., Shimazawa M., Hara H. (2014). Damage of photoreceptor-derived cells in culture induced by light emitting diode-derived blue light. *Scientific Reports*.

[11] Jain G. K., Warsi M. H., Nirmal J. (2012). Therapeutic stratagems for vascular degenerative disorders of the posterior eye. *Drug Discovery Today*.

[12] Ahsan H. (2015). Diabetic retinopathy—biomolecules and multiple pathophysiology. *Diabetes and Metabolic Syndrome: Clinical Research and Reviews*.

[13] Cabrera M. P., Chihuailaf R. H. (2011). Antioxidants and the integrity of ocular tissues. *Veterinary Medicine International*.

[14] Handa J. T. (2012). How does the macula protect itself from oxidative stress?. *Molecular Aspects of Medicine*.

[15] Jarrett S. G., Boulton M. E. (2012). Consequences of oxidative stress in age-related macular degeneration. *Molecular Aspects of Medicine*.

[16] Hollyfield J. G., Bonilha V. L., Rayborn M. E. (2008). Oxidative damage-induced inflammation initiates age-related macular degeneration. *Nature Medicine*.

[17] Chew E. Y., Clemons T. E., Agrón E., Launer L. J., Grodstein F., Bernstein P. S. (2015). Effect of omega-3 fatty acids, lutein/zeaxanthin, or other nutrient supplementation on cognitive function: the AREDS2 randomized clinical trial. *The Journal of the American Medical Association*.

[18] Babizhayev M. A. (2010). New concept in nutrition for the maintenance of the aging eye redox regulation and therapeutic treatment of cataract disease; Synergism of natural antioxidant imidazole-containing amino acid-based compounds, chaperone, and glutathione boosting agents: a systemic perspective on aging and longevity emerged from studies in humans. *American Journal of Therapeutics*.

[19] Berson E. L., Rosner B., Sandberg M. A. (2010). Clinical trial of lutein in patients with retinitis pigmentosa receiving vitamin A. *Archives of Ophthalmology*.

[20] Wong W. T., Kam W., Cunningham D. (2010). Treatment of geographic atrophy by the topical administration of OT-551: results of a phase II clinical trial. *Investigative Ophthalmology & Visual Science*.

[21] Itoh K., Mimura J., Yamamoto M. (2010). Discovery of the negative regulator of Nrf2, keap1: a historical overview. *Antioxidants and Redox Signaling*.

[22] Suzuki T., Motohashi H., Yamamoto M. (2013). Toward clinical application of the Keap1-Nrf2 pathway. *Trends in Pharmacological Sciences*.

[23] Ruiz S., Pergola P. E., Zager R. A., Vaziri N. D. (2013). Targeting the transcription factor Nrf2 to ameliorate oxidative stress and inflammation in chronic kidney disease. *Kidney International*.

[24] Wardyn J. D., Ponsford A. H., Sanderson C. M. (2015). Dissecting molecular cross-talk between Nrf2 and NF-*κ*B response pathways. *Biochemical Society Transactions*.

[25] Rahman I. (2006). Antioxidant therapies in COPD. *International Journal of Chronic Obstructive Pulmonary Disease*.

[26] Suzuki M., Betsuyaku T., Ito Y. (2008). Down-regulated NF-E2-related factor 2 in pulmonary macrophages of aged smokers and patients with chronic obstructive pulmonary disease. *American Journal of Respiratory Cell and Molecular Biology*.

[27] Zhong Q., Mishra M., Kowluru R. A. (2013). Transcription factor Nrf2-mediated antioxidant defense system in the development of diabetic retinopathy. *Investigative Ophthalmology & Visual Science*.

[28] Lambros M. L., Plafker S. M. (2016). Oxidative stress and the Nrf2 anti-oxidant transcription factor in age-related macular degeneration. *Advances in Experimental Medicine and Biology*.

[29] Itoh K., Chiba T., Takahashi S. (1997). An Nrf2/small Maf heterodimer mediates the induction of phase II detoxifying enzyme genes through antioxidant response elements. *Biochemical and Biophysical Research Communications*.

[30] Chan K., Lu R., Chang J. C., Kan Y. W. (1996). NRF2, a member of the NFE2 family of transcription factors, is not essential for murine erythropoiesis, growth, and development. *Proceedings of the National Academy of Sciences of the United States of America*.

[31] Zhao Z., Chen Y., Wang J. (2011). Age-related retinopathy in NRF2-deficient mice. *PLoS ONE*.

[32] Cano M., Thimmalappula R., Fujihara M. (2010). Cigarette smoking, oxidative stress, the anti-oxidant response through Nrf2 signaling, and Age-related Macular Degeneration. *Vision Research*.

[33] Uno K., Prow T. W., Bhutto I. A. (2010). Role of Nrf2 in retinal vascular development and the vaso-obliterative phase of oxygen-induced retinopathy. *Experimental Eye Research*.

[34] Himori N., Yamamoto K., Maruyama K. (2013). Critical role of Nrf2 in oxidative stress-induced retinal ganglion cell death. *Journal of Neurochemistry*.

[35] Xu Z., Wei Y., Gong J. (2014). NRF2 plays a protective role in diabetic retinopathy in mice. *Diabetologia*.

[36] Nagai N., Thimmulappa R. K., Cano M. (2009). Nrf2 is a critical modulator of the innate immune response in a model of uveitis. *Free Radical Biology and Medicine*.

[37] Wei Y., Gong J., Yoshida T. (2011). Nrf2 has a protective role against neuronal and capillary degeneration in retinal ischemia-reperfusion injury. *Free Radical Biology and Medicine*.

[38] Wakabayashi N., Itoh K., Wakabayashi J. (2003). Keap1-null mutation leads to postnatal lethality due to constitutive Nrf2 activation. *Nature Genetics*.

[39] Okawa H., Motohashi H., Kobayashi A., Aburatani H., Kensler T. W., Yamamoto M. (2006). Hepatocyte-specific deletion of the keap1 gene activates Nrf2 and confers potent resistance against acute drug toxicity. *Biochemical and Biophysical Research Communications*.

[40] Kensler T. W., Egner P. A., Agyeman A. S. (2013). Keap1-Nrf2 signaling: a target for cancer prevention by sulforaphane. *Topics in Current Chemistry*.

[41] Takaya K., Suzuki T., Motohashi H. (2012). Validation of the multiple sensor mechanism of the Keap1-Nrf2 system. *Free Radical Biology and Medicine*.

[42] Lu M. C., Ji J. A., Jiang Z. Y., You Q. D. (2016). The Keap1-Nrf2-ARE pathway as a potential preventive and therapeutic target: an update. *Medicinal Research Reviews*.

[43] Houghton C. A., Fassett R. G., Coombes J. S. (2016). Sulforaphane and other nutrigenomic Nrf2 activators: can the clinician's expectation be matched by the reality?. *Oxidative Medicine and Cellular Longevity*.

[44] Deeks E. D. (2016). Dimethyl fumarate: a review in relapsing-remitting MS. *Drugs*.

[45] Liby K. T., Sporn M. B. (2012). Synthetic oleanane triterpenoids: multifunctional drugs with a broad range of applications for prevention and treatment of chronic disease. *Pharmacological Reviews*.

[46] Liby K. T., Yore M. M., Sporn M. B. (2007). Triterpenoids and rexinoids as multifunctional agents for the prevention and treatment of cancer. *Nature Reviews Cancer*.

[47] Pergola P. E., Raskin P., Toto R. D. (2011). Bardoxolone methyl and kidney function in CKD with type 2 diabetes. *The New England Journal of Medicine*.

[48] de Zeeuw D., Akizawa T., Audhya P. (2013). Bardoxolone methyl in type 2 diabetes and stage 4 chronic kidney disease. *The New England Journal of Medicine*.

[49] Chin M. P., Reisman S. A., Bakris G. L. (2014). Mechanisms contributing to adverse cardiovascular events in patients with type 2 diabetes mellitus and stage 4 chronic kidney disease treated with bardoxolone methyl. *American Journal of Nephrology*.

[50] Abboud H. E. (2013). Synthetic oleanane triterpenoids: magic bullets or not?. *Kidney International*.

[51] Pitha-Rowe I., Liby K., Royce D., Sporn M. (2009). Synthetic triterpenoids attenuate cytotoxic retinal injury: cross-talk between Nrf2 and PI3K/AKT signaling through inhibition of the lipid phosphatase PTEN. *Investigative Ophthalmology and Visual Science*.

[52] Sussan T. E., Rangasamy T., Blake D. J. (2009). Targeting Nrf2 with the triterpenoid CDDO-imidazolide attenuates cigarette smoke-induced emphysema and cardiac dysfunction in mice. *Proceedings of the National Academy of Sciences of the United States of America*.

[53] Nakagami Y., Masuda K., Hatano E. (2015). Novel Nrf2 activators from microbial transformation products inhibit blood-retinal barrier permeability in rabbits. *British Journal of Pharmacology*.

[54] Nakagami Y., Hatano E., Inoue T., Yoshida K., Kondo M., Terasaki H. (2016). Cytoprotective effects of a novel Nrf2 activator, RS9, in rhodopsin Pro347Leu rabbits. *Current Eye Research*.

[55] Shen H.-H., Chan E. C., Lee J. H. (2015). Nanocarriers for treatment of ocular neovascularization in the back of the eye: new vehicles for ophthalmic drug delivery. *Nanomedicine*.

[56] Kuno N., Fujii S. (2010). Biodegradable intraocular therapies for retinal disorders: progress to date. *Drugs and Aging*.

[57] Wells G. (2015). Peptide and small molecule inhibitors of the Keap1-Nrf2 protein-protein interaction. *Biochemical Society Transactions*.

[58] Abed D. A., Goldstein M., Albanyan H., Jin H., Hu L. (2015). Discovery of direct inhibitors of Keap1-Nrf2 protein-protein interaction as potential therapeutic and preventive agents. *Acta Pharmaceutica Sinica B*.

[59] Tong K. I., Padmanabhan B., Kobayashi A. (2007). Different electrostatic potentials define ETGE and DLG motifs as hinge and latch in oxidative stress response. *Molecular and Cellular Biology*.

[60] Hu L., Magesh S., Chen L. (2013). Discovery of a small-molecule inhibitor and cellular probe of Keap1-Nrf2 protein-protein interaction. *Bioorganic and Medicinal Chemistry Letters*.

[61] Shimozono R., Asaoka Y., Yoshizawa Y. (2013). Nrf2 activators attenuate the progression of nonalcoholic steatohepatitis-related fibrosis in a dietary rat models. *Molecular Pharmacology*.

[62] Canning P., Bullock A. N. (2014). New strategies to inhibit KEAP1 and the Cul3-based E3 ubiquitin ligases. *Biochemical Society Transactions*.

[63] Kobayashi A., Kang M.-I., Okawa H. (2004). Oxidative stress sensor Keap1 functions as an adaptor for Cul3-based E3 ligase to regulate proteasomal degradation of Nrf2. *Molecular and Cellular Biology*.

[64] Furukawa M., Xiong Y. (2005). BTB protein keap1 targets antioxidant transcription factor Nrf2 for ubiquitination by the cullin 3-Roc1 ligase. *Molecular and Cellular Biology*.

[65] Itoh K., Wakabayashi N., Katoh Y., Ishii T., O'Connor T., Yamamoto M. (2003). Keap1 regulates both cytoplasmic-nuclear shuttling and degradation of Nrf2 in response to electrophiles. *Genes to Cells*.

[66] Solano E. C. R., Kornbrust D. J., Beaudry A., Foy J. W.-D., Schneider D. J., Thompson J. D. (2014). Toxicological and pharmacokinetic properties of QPI-1007, a chemically modified synthetic siRNA targeting caspase 2 mRNA, following intravitreal injection. *Nucleic Acid Therapeutics*.

[67] Morgan-Warren P. J., O’Neill J., De Cogan F. (2016). SiRNA-mediated knockdown of the mTOR inhibitor RTP801 promotes retinal ganglion cell survival and axon elongation by direct and indirect mechanisms. *Investigative Ophthalmology and Visual Science*.

[68] Hayes J. D., McMahon M. (2009). NRF2 and KEAP1 mutations: permanent activation of an adaptive response in cancer. *Trends in Biochemical Sciences*.

